# 3-Carbamoylquinoxalin-1-ium chloride

**DOI:** 10.1107/S1600536811052457

**Published:** 2011-12-10

**Authors:** James K. Harper, Gary Strobel, Atta M. Arif

**Affiliations:** a University of Central Florida, Department of Chemistry, 4000 Central Florida Blvd., Orlando, FL 32816, USA; b Montana State University, Department of Plant Sciences and Plant Patology, Bozeman, MT 59717, USA; c University of Utah, Department of Chemistry, 315 S. 1400 E. Rm. 2020, Salt Lake City, UT 84112, USA

## Abstract

The title compound, C_9_H_8_N_3_O^+^·Cl^−^, was isolated from a liquid culture of *streptomyces* sp. In the cation, the ring system makes a dihedral angle of 0.2 (2)° with the amide group. The protonation creating the cation occurs at ome of the N atoms in the quinoxaline ring system. In the crystal, the ions are linked through N—H⋯O and N—H⋯Cl hydrogen bonds, forming a two-dimensional network parallel to (10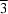).

## Related literature

For a description of the bioactivity and mode of action of compounds containing the quinoxaline moiety, see: Bailly *et al.* (1999 ▶); May *et al.* (2004 ▶); Mollegaard *et al.* (2000 ▶); Waring (1993 ▶). For crystal structures of the mol­ecules triostin A, echinomycin and their derivatives, which all contain two quinoxalines, see: Hossain *et al.* (1982 ▶); Sheldrick *et al.* (1984 ▶, 1995 ▶); Viswamitra *et al.* (1981 ▶); Wang *et al.* (1984 ▶); Ughetto *et al.* (1985 ▶). For a description of the Streptomycete producing the title compound, see: Castillo *et al.* (2003 ▶).
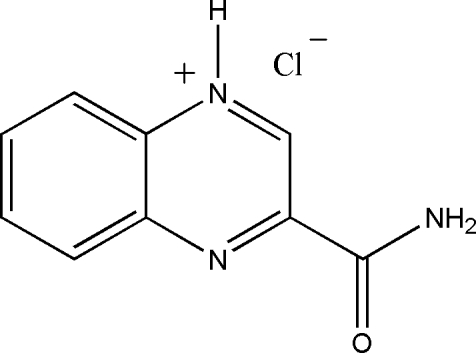


## Experimental

### 

#### Crystal data

C_9_H_8_N_3_O^+^·Cl^−^
*M* *_r_* = 209.63
Monoclinic, 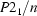

*a* = 5.6476 (2) Å
*b* = 15.1045 (9) Å
*c* = 11.2556 (6) Å
β = 99.993 (3)°
*V* = 945.58 (8) Å^3^
*Z* = 4
Mo *K*α radiation
μ = 0.37 mm^−1^
*T* = 150 K
0.25 × 0.20 × 0.08 mm

#### Data collection

Nonius KappaCCD diffractometer
Absorption correction: multi-scan (*DENZO-SMN*; Otwinowski & Minor, 1997 ▶) *T*_min_ = 0.913, *T*_max_ = 0.971
3671 measured reflections
2147 independent reflections
1798 reflections with *I* > 2σ(*I*)
*R*_int_ = 0.018

#### Refinement

*R*[*F* ^2^ > 2σ(*F* ^2^)] = 0.034
*wR*(*F* ^2^) = 0.085
*S* = 1.05
2147 reflections
160 parameters
All H-atom parameters refined
Δρ_max_ = 0.25 e Å^−3^
Δρ_min_ = −0.24 e Å^−3^

### 

Data collection: *COLLECT* (Nonius, 1998 ▶); cell refinement: *DENZO-SMN* (Otwinowski & Minor, 1997 ▶); data reduction: *DENZO-SMN*; program(s) used to solve structure: *SIR97* (Altomare *et al.*, 1999 ▶); program(s) used to refine structure: *SHELXL97* (Sheldrick, 2008 ▶); molecular graphics: *WinGX* (Farrugia, 1999 ▶) and *ORTEP-3* (Farrugia, 1997 ▶); software used to prepare material for publication: *SHELXL97*.

## Supplementary Material

Crystal structure: contains datablock(s) I, global. DOI: 10.1107/S1600536811052457/lh5381sup1.cif

Structure factors: contains datablock(s) I. DOI: 10.1107/S1600536811052457/lh5381Isup2.hkl

Supplementary material file. DOI: 10.1107/S1600536811052457/lh5381Isup3.cml

Additional supplementary materials:  crystallographic information; 3D view; checkCIF report

## Figures and Tables

**Table 1:** Hydrogen-bond geometry (Å, °)

*D*—H⋯*A*	*D*—H	H⋯*A*	*D*⋯*A*	*D*—H⋯*A*
N1—H1*A*⋯O1^i^	0.86 (2)	2.04 (2)	2.9008 (17)	173.5 (17)
N1—H1*B*⋯Cl1	0.90 (2)	2.44 (2)	3.2590 (13)	152.0 (17)
N3—H3*N*⋯Cl1^ii^	0.94 (2)	2.02 (2)	2.9501 (13)	169.8 (15)

Symmetry codes: (i) 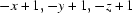; (ii) 
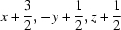
.

**Fig. 1.**
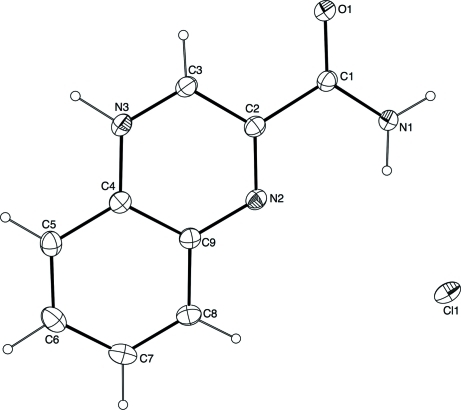
Molecular structure of the title compound. Displacement ellipsoids are shown at the 50% probability level on non-hydrogen atoms.

**Fig. 2.**
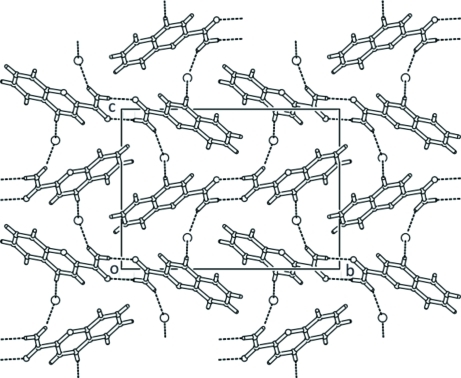
Part of the crystal structure viewed along the a axis. The dashed lines indicate N—H···O and N—H···Cl hydrogen bonds.

## supplementary crystallographic information

### Comment

The quinoxaline ring is an essential component of the DNA intercalators echinomycin and triostin A. The two quinoxaline rings present in each of these compounds bind the minor groove of double stranded DNA and thereby inhibit RNA synthesis (Bailly *et al.*, 1999; May *et al.*, 2004; Mollegaard *et al.*, 2000; Waring, 1993). Presently, the quinoxaline ring has been characterized crystallographically only as part of a significantly larger molecular assembly (Hossain *et al.*, 1982; Sheldrick *et al.*,1984; Sheldrick *et al.*, 1995; Viswamitra *et al.*, 1981; Wang *et al.*, 1984; Ughetto *et al.*, 1985). Accordingly, the resolution of the quinoxaline moieties currently established is relatively low. Here, characterization of a simpler quinoxaline ring system provides a higher resolution dataset for a compound having a substitution pattern identical to that found in the quinoxaline antibiotics. The conformation about the C1—C2 bond in the title compound is shown in Figure 1 and matches that reported for triostin A and echinomycin. Molecules in the crystal are linked through N1—H···O1^i^ (see Table 1 for symmetry codes) hydrogen bonds as well as N1—H···Cl···H—N3 interaction. The structure viewed along the *a* axis is shown in figure 2.

### Experimental

The title compound was obtained by liquid-liquid extraction (CH_2_Cl_2_/H_2_O) of a culture of an endophytic *Streptomyces* sp. described elsewhere (Castillo *et al.*, 2003). A crystal was grown by slow evaporation of a 1:1 mix of CHCl_3_:MeOH

### Refinement

All H atoms were refined independently with isotropic displacement parameters.

### Crystal data

**Table:**

C_9_H_8_N_3_O^+^·Cl^−^	*F*(000) = 432
*M**_r_* = 209.63	*D*_x_ = 1.473 Mg m^−^^3^
Monoclinic, *P*2_1_/*n*	Mo *K*α radiation, λ = 0.71073 Å
Hall symbol: -P 2yn	Cell parameters from 1998 reflections
*a* = 5.6476 (2) Å	θ = 1.0–27.5°
*b* = 15.1045 (9) Å	µ = 0.37 mm^−^^1^
*c* = 11.2556 (6) Å	*T* = 150 K
β = 99.993 (3)°	Plate, pale yellow
*V* = 945.58 (8) Å^3^	0.25 × 0.20 × 0.08 mm
*Z* = 4	

### Data collection

**Table:**

Nonius KappaCCD diffractometer	2147 independent reflections
Radiation source: fine-focus sealed tube	1798 reflections with *I* > 2σ(*I*)
graphite	*R*_int_ = 0.018
φ and ω scans	θ_max_ = 27.5°, θ_min_ = 3.9°
Absorption correction: multi-scan (*DENZO-SMN*; Otwinowski & Minor, 1997)	*h* = −7→7
*T*_min_ = 0.913, *T*_max_ = 0.971	*k* = −18→19
3671 measured reflections	*l* = −14→14

### Refinement

**Table:**

Refinement on *F*^2^	Secondary atom site location: difference Fourier map
Least-squares matrix: full	Hydrogen site location: inferred from neighbouring sites
*R*[*F*^2^ > 2σ(*F*^2^)] = 0.034	All H-atom parameters refined
*wR*(*F*^2^) = 0.085	*w* = 1/[σ^2^(*F*_o_^2^) + (0.0397*P*)^2^ + 0.2499*P*] where *P* = (*F*_o_^2^ + 2*F*_c_^2^)/3
*S* = 1.05	(Δ/σ)_max_ < 0.001
2147 reflections	Δρ_max_ = 0.25 e Å^−^^3^
160 parameters	Δρ_min_ = −0.24 e Å^−^^3^
0 restraints	Extinction correction: *SHELXL97* (Sheldrick, 2008), Fc^*^=kFc[1+0.001xFc^2^λ^3^/sin(2θ)]^-1/4^
Primary atom site location: structure-invariant direct methods	Extinction coefficient: 0.012 (4)

### Special details

**Table:**

Experimental. The program *DENZO-SMN* (Otwinowski & Minor, 1997) uses a scaling algorithm that effectively corrects for absorption effects. High redundancy data were used in the scaling program hence the 'multi-scan' code word was used. No transmission coefficients are available from the program (only scale factors for each frame). The scale factors in the experimental table are calculated from the 'size' command in the *SHELXL97* input file.
Geometry. All s.u.'s (except the s.u. in the dihedral angle between two l.s. planes) are estimated using the full covariance matrix. The cell s.u.'s are taken into account individually in the estimation of s.u.'s in distances, angles and torsion angles; correlations between s.u.'s in cell parameters are only used when they are defined by crystal symmetry. An approximate (isotropic) treatment of cell s.u.'s is used for estimating s.u.'s involving l.s. planes.
Refinement. Refinement of *F*^2^ against ALL reflections. The weighted *R*-factor *wR* and goodness of fit *S* are based on *F*^2^, conventional *R*-factors *R* are based on *F*, with *F* set to zero for negative *F*^2^. The threshold expression of *F*^2^ > σ(*F*^2^) is used only for calculating *R*-factors(gt) *etc*. and is not relevant to the choice of reflections for refinement. *R*-factors based on *F*^2^ are statistically about twice as large as those based on *F*, and *R*-factors based on ALL data will be even larger.

### Fractional atomic coordinates and isotropic or equivalent isotropic displacement parameters (Å^2^)

**Table:**

	*x*	*y*	*z*	*U*_iso_*/*U*_eq_	
Cl1	0.08949 (6)	0.30272 (3)	0.19526 (3)	0.03255 (15)	
O1	0.76925 (19)	0.43611 (7)	0.55896 (10)	0.0336 (3)	
N1	0.4423 (2)	0.39023 (9)	0.42509 (12)	0.0269 (3)	
N2	0.6771 (2)	0.23505 (8)	0.39555 (10)	0.0234 (3)	
N3	1.1403 (2)	0.21019 (8)	0.51784 (11)	0.0248 (3)	
C1	0.6625 (3)	0.38080 (9)	0.48828 (13)	0.0247 (3)	
C2	0.7884 (2)	0.29539 (9)	0.46906 (12)	0.0234 (3)	
C3	1.0240 (3)	0.28321 (10)	0.53318 (13)	0.0254 (3)	
C4	1.0391 (2)	0.14589 (9)	0.43990 (12)	0.0237 (3)	
C5	1.1680 (3)	0.06898 (10)	0.42012 (14)	0.0289 (3)	
C6	1.0562 (3)	0.00651 (11)	0.34196 (14)	0.0338 (4)	
C7	0.8153 (3)	0.01774 (11)	0.28407 (14)	0.0331 (4)	
C8	0.6884 (3)	0.09223 (10)	0.30207 (13)	0.0276 (3)	
C9	0.7997 (2)	0.15942 (9)	0.37975 (12)	0.0229 (3)	
H1A	0.370 (3)	0.4397 (13)	0.4318 (16)	0.036 (5)*	
H1B	0.381 (4)	0.3498 (15)	0.3699 (19)	0.051 (6)*	
H3	1.102 (3)	0.3227 (12)	0.5857 (17)	0.035 (5)*	
H3N	1.290 (3)	0.2011 (11)	0.5682 (17)	0.034 (5)*	
H5	1.324 (3)	0.0634 (12)	0.4615 (16)	0.035 (5)*	
H6	1.139 (3)	−0.0467 (12)	0.3273 (15)	0.032 (4)*	
H7	0.738 (3)	−0.0276 (13)	0.2325 (17)	0.041 (5)*	
H8	0.523 (3)	0.1023 (10)	0.2602 (15)	0.027 (4)*	

### Atomic displacement parameters (Å^2^)

**Table:**

	*U*^11^	*U*^22^	*U*^33^	*U*^12^	*U*^13^	*U*^23^
Cl1	0.0270 (2)	0.0419 (2)	0.0258 (2)	−0.00693 (15)	−0.00344 (14)	−0.00243 (15)
O1	0.0323 (6)	0.0264 (5)	0.0365 (6)	0.0033 (4)	−0.0097 (5)	−0.0054 (5)
N1	0.0258 (6)	0.0235 (6)	0.0284 (7)	0.0029 (5)	−0.0037 (5)	−0.0018 (5)
N2	0.0240 (6)	0.0249 (6)	0.0205 (6)	−0.0011 (5)	0.0015 (5)	0.0019 (5)
N3	0.0217 (6)	0.0290 (6)	0.0225 (6)	0.0013 (5)	0.0000 (5)	0.0016 (5)
C1	0.0263 (7)	0.0231 (7)	0.0228 (7)	−0.0001 (6)	−0.0011 (5)	0.0023 (6)
C2	0.0235 (7)	0.0251 (7)	0.0212 (7)	−0.0014 (5)	0.0030 (5)	0.0016 (5)
C3	0.0244 (7)	0.0268 (7)	0.0232 (7)	−0.0009 (6)	−0.0007 (6)	−0.0007 (6)
C4	0.0253 (7)	0.0258 (7)	0.0204 (7)	−0.0011 (5)	0.0051 (5)	0.0029 (5)
C5	0.0286 (8)	0.0315 (8)	0.0274 (8)	0.0053 (6)	0.0075 (6)	0.0033 (6)
C6	0.0433 (9)	0.0288 (8)	0.0321 (8)	0.0056 (7)	0.0144 (7)	−0.0001 (7)
C7	0.0428 (9)	0.0303 (8)	0.0279 (8)	−0.0050 (7)	0.0102 (7)	−0.0071 (7)
C8	0.0296 (8)	0.0314 (8)	0.0220 (7)	−0.0046 (6)	0.0052 (6)	−0.0018 (6)
C9	0.0257 (7)	0.0246 (7)	0.0189 (7)	−0.0008 (6)	0.0050 (5)	0.0022 (5)

### Geometric parameters (Å, °)

**Table:**

O1—C1	1.2361 (17)	C3—H3	0.900 (19)
N1—C1	1.3285 (18)	C4—C5	1.409 (2)
N1—H1A	0.86 (2)	C4—C9	1.4178 (19)
N1—H1B	0.90 (2)	C5—C6	1.368 (2)
N2—C2	1.3154 (18)	C5—H5	0.928 (17)
N2—C9	1.3635 (18)	C6—C7	1.413 (2)
N3—C3	1.3104 (19)	C6—H6	0.957 (18)
N3—C4	1.3660 (19)	C7—C8	1.368 (2)
N3—H3N	0.94 (2)	C7—H7	0.95 (2)
C1—C2	1.5066 (19)	C8—C9	1.414 (2)
C2—C3	1.411 (2)	C8—H8	0.980 (16)
			
C1—N1—H1A	117.3 (12)	N3—C4—C9	117.53 (13)
C1—N1—H1B	120.9 (13)	C5—C4—C9	121.29 (13)
H1A—N1—H1B	121.2 (18)	C6—C5—C4	118.49 (15)
C2—N2—C9	117.67 (12)	C6—C5—H5	123.5 (11)
C3—N3—C4	121.30 (13)	C4—C5—H5	118.0 (11)
C3—N3—H3N	117.5 (10)	C5—C6—C7	120.93 (15)
C4—N3—H3N	120.9 (10)	C5—C6—H6	120.4 (10)
O1—C1—N1	125.36 (13)	C7—C6—H6	118.6 (10)
O1—C1—C2	118.78 (12)	C8—C7—C6	121.19 (15)
N1—C1—C2	115.85 (12)	C8—C7—H7	118.8 (11)
N2—C2—C3	122.45 (13)	C6—C7—H7	120.0 (11)
N2—C2—C1	119.85 (12)	C7—C8—C9	119.58 (14)
C3—C2—C1	117.69 (12)	C7—C8—H8	122.4 (9)
N3—C3—C2	119.48 (13)	C9—C8—H8	118.0 (9)
N3—C3—H3	116.4 (12)	N2—C9—C8	120.04 (13)
C2—C3—H3	124.2 (12)	N2—C9—C4	121.50 (13)
N3—C4—C5	121.17 (13)	C8—C9—C4	118.46 (13)
			
C9—N2—C2—C3	−1.7 (2)	C9—C4—C5—C6	−0.6 (2)
C9—N2—C2—C1	179.05 (12)	C4—C5—C6—C7	−1.3 (2)
O1—C1—C2—N2	179.06 (13)	C5—C6—C7—C8	1.6 (2)
N1—C1—C2—N2	−1.63 (19)	C6—C7—C8—C9	0.1 (2)
O1—C1—C2—C3	−0.2 (2)	C2—N2—C9—C8	179.78 (13)
N1—C1—C2—C3	179.12 (13)	C2—N2—C9—C4	−0.14 (19)
C4—N3—C3—C2	0.7 (2)	C7—C8—C9—N2	178.09 (13)
N2—C2—C3—N3	1.5 (2)	C7—C8—C9—C4	−2.0 (2)
C1—C2—C3—N3	−179.26 (12)	N3—C4—C9—N2	2.22 (19)
C3—N3—C4—C5	177.53 (13)	C5—C4—C9—N2	−177.80 (12)
C3—N3—C4—C9	−2.5 (2)	N3—C4—C9—C8	−177.70 (12)
N3—C4—C5—C6	179.33 (13)	C5—C4—C9—C8	2.3 (2)

### Hydrogen-bond geometry (Å, °)

**Table:**

*D*—H···*A*	*D*—H	H···*A*	*D*···*A*	*D*—H···*A*
N1—H1A···O1^i^	0.86 (2)	2.04 (2)	2.9008 (17)	173.5 (17)
N1—H1B···Cl1	0.90 (2)	2.44 (2)	3.2590 (13)	152.0 (17)
N3—H3N···Cl1^ii^	0.94 (2)	2.02 (2)	2.9501 (13)	169.8 (15)

Symmetry codes: (i) −*x*+1, −*y*+1, −*z*+1; (ii) *x*+3/2, −*y*+1/2, *z*+1/2.
